# Low-voltage magnetoelectric coupling in membrane heterostructures

**DOI:** 10.1126/sciadv.abh2294

**Published:** 2021-11-12

**Authors:** Shane Lindemann, Julian Irwin, Gi-Yeop Kim, Bo Wang, Kitae Eom, Jianjun Wang, Jiamian Hu, Long-Qing Chen, Si-Young Choi, Chang-Beom Eom, Mark S. Rzchowski

**Affiliations:** 1 Department of Materials Science and Engineering, University of Wisconsin-Madison, Madison, WI 53706, USA.; 2 Department of Physics, University of Wisconsin-Madison, Madison, WI 53706, USA.; 3 Department of Materials Science and Engineering, Pohang University of Science and Technology, Pohang, Gyeongbuk 37673, Korea.; 4 Department of Materials Science and Engineering, Pennsylvania State University, University Park, PA 16802, USA.

## Abstract

Strain-mediated magnetoelectric (ME) coupling in ferroelectric (FE)/ferromagnetic (FM) heterostructures offers a unique opportunity for both fundamental scientific research and low-power multifunctional devices. Relaxor-FEs, such as (1 − *x*)Pb(Mg_1/3_Nb_2/3_)O_3_-(*x*)PbTiO_3_ (PMN-*x*PT), are ideal FE layer candidates because of their giant piezoelectricity. However, thin films of PMN-PT suffer from substrate clamping, which substantially reduces piezoelectric in-plane strains. Here, we demonstrate low-voltage ME coupling in an all-thin-film heterostructure that uses the anisotropic strains induced by the (011) orientation of PMN-PT. We completely remove PMN-PT films from their substrate and couple with FM Ni overlayers to create membrane PMN-PT/Ni heterostructures showing 90° Ni magnetization rotation with 3 V PMN-PT bias, much less than the bulk PMN-PT ~100-V requirement. Scanning transmission electron microscopy and phase-field simulations clarify the membrane response. These results provide a crucial step toward understanding the microstructural behavior of PMN-PT thin films for use in piezo-driven ME heterostructures.

## Teaser

Membrane-based heterostructure, verified experimentally and theoretically, opens possibiliies for low-power magnetoelectrics.

## INTRODUCTION

Electric field control of magnetism, also known as converse magnetoelectric (ME) coupling, in ferromagnetic (FM)/ferroelectric (FE) composites is of considerable interest because of the potential for its development as next-generation memory storage and sensing technologies (*1*, *2*). Of particular interest for use as the FE layer are relaxor-ferroelectrics, such as (1 − *x*)Pb(Mg_1/3_Nb_2/3_)O_3_-(*x*)PbTiO_3_ (PMN-PT), which show a large piezoelectric response for compositions near a morphotropic phase boundary (*x* = ~35% for PMN-PT) (*3*). By coupling the relaxor-ferroelectric with an FM containing large magnetostriction, converse ME coupling is achieved through transfer of the voltage-induced strain from the FE layer into the FM layer that can result in strain-mediated control of in-plane magnetic anisotropy (*4*–*6*), tunneling magnetoresistance (*7*), FM resonance (*8*), and conductivity (*9*).

**Fig. 1.**
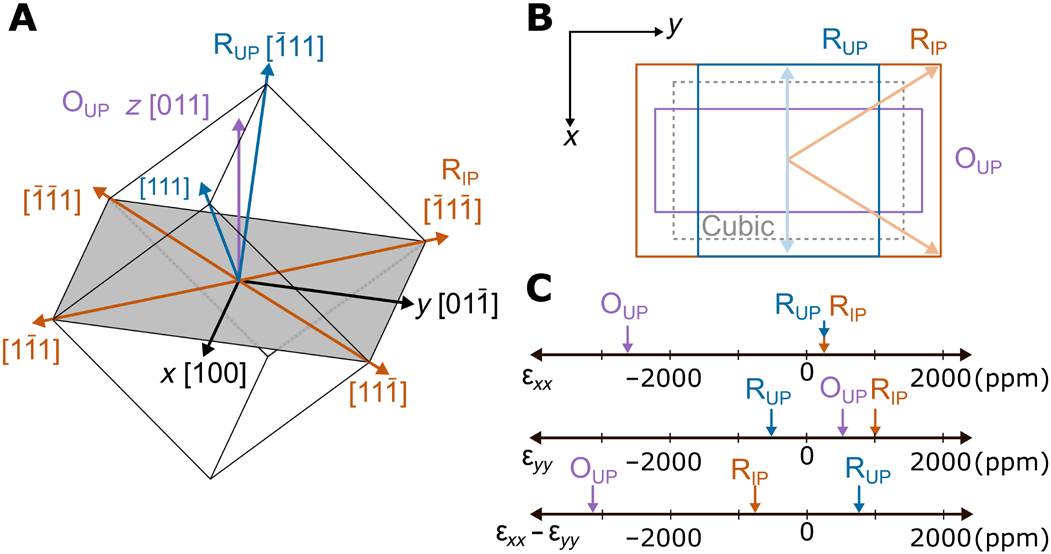
Anisotropic strain in (011)-oriented PMN-PT. (**A**) Cartesian coordinates *x*, *y*, and *z* are defined to be the crystal [100],$\left[01\bar{1}\right]$, and [011] directions, respectively. Polarization directions in (011)-oriented PMN-PT unit cell, grouped into rhombohedral in-plane (R_IP_; orange), rhombohedral up (R_UP_; blue), and orthorhombic up (O_UP_; purple). Rhombohedral down (R_DOWN_) and orthorhombic down (O_DOWN_) are not shown but are, respectively, R_UP_ and O_UP_ mirrored about the *xy* plane. The in-plane cut through the unit cell (shaded gray area) is rectangular with sides of length $a\sqrt{2}$by *a*, where *a* is the lattice parameter. (**B**) Electrostrictive deformations (not to scale) of the unit cell for the cubic (zero FE polarization), R_IP_, R_UP_, and O_UP_ polarization groups. The down deformations are identical to up. In-plane projections of polarization vectors are shown for R_IP_ (light orange) and R_UP_ (light blue). (**C**) Plots of linear electrostriction strains ε*_xx_* and ε*_yy_* and the anisotropic strain ε*_xx_* − ε*_yy_* for R_IP_, R_UP_, and O_UP_ polarization groups. Numerical values are in table S1.

For many strain-mediated ME coupling applications, including rotation of the in-plane magnetization of a coupled FM, anisotropic in-plane strains are required. Therefore, the abovementioned studies (*4*–*7*, *9*) used (011)-oriented bulk single crystals of PMN-PT that develop large anisotropic in-plane strains under an electric field. Fig. 1A shows the crystal geometry for the (011) orientation. The *x* = 30% composition of PMN-PT is rhombohedral (R) with spontaneous polarization along the 〈111〉 directions (*10*). These polarization directions can be grouped as rhombohedral up (R_UP_), rhombohedral in-plane (R_IP_), and rhombohedral down (R_DOWN_) (*4*). In addition, applying a large electric field can stabilize a polarization parallel to the applied field direction [011], giving the crystal an orthorhombic (O) up (O_UP_; purple) symmetry (*11*). Each of these polarization groups results in average strained unit cells projected into the (011) plane as shown in Fig. 1B, with the unstrained cubic cell (dashed lines) as a reference. This uses equal-weight averaging of each polarization vector present in the group. The normal strains associated with each polarization group may be calculated using the PMN-PT electrostriction tensor (see table S1), which has been measured in bulk (*12*). Figure 1C shows the normal strains ε*_xx_* and ε*_yy_* along the *x* and *y* directions, respectively, as well as the anisotropic in-plane strain ε*_xx_* − ε*_yy_* for all three polarization groups with the same magnitude of polarization, again averaged over all FE domains in the polarization group. Studies using bulk PMN-PT primarily used 71°/109° permanent switching between R_UP_ and the metastable R_IP_ polarization states, although, as seen in Fig. 1C, inducing polarization rotation from R to O can result in even larger strain anisotropy. The R-to-O transition is often deemed undesirable in bulk studies because of the large required voltage as well as it being a nonpermanent effect, i.e., the strains will relax once the voltage bias is removed. In thin films, however, the high electric fields needed for polarization rotation between the R and O directions can be more easily achieved and plays a large role in this study.

The drive toward low-power ME devices (*13*, *14*), as well as development of micro- and nanoelectromechanical systems, has prompted the study of relaxor-ferroelectric thin films (*15*–*17*). Upon reduction to thin-film dimensions, relaxor-ferroelectrics suffer a large reduction in piezoelectricity owing to mechanical clamping by a passive substrate (*18*–*23*). Such a limitation presents a substantial challenge toward successful integration of relaxor-ferroelectric thin films in high-performance devices. Several methods have been used to reduce clamping in FE thin films including growth directly on flexible substrates (*24*), micromachining into bendable cantilevers (*25*), fine lithographic patterning (*26*–*28*), micromachining (*29*, *30*), and direct ME coupling for magnetic field sensing in thin-film cantilevers (*31*, *32*). However, obtaining the largest strain-mediated ME response in thin-film heterostructures necessitates complete removal of the substrate to allow a free piezoresponse.

Many device concepts based on the piezo-driven ME effect rely on the use of (011)-oriented PMN-PT thin films because of the abovementioned demonstrations involving bulk PMN-PT. Up to now, however, demonstration of piezo-driven ME coupling in all-thin-film FE/FM heterostructures has been hindered by the issue of substrate clamping. In this study, we overcome the clamping issue and provide demonstration of low-voltage strain-mediated ME coupling in an all-thin-film heterostructure that only relies on the anisotropic strains inherent to the (011) orientation of PMN-PT. We fabricate unclamped (011)-oriented PMN-PT thin-film membranes, through release from a rigid substrate using a sacrificial etching layer, and couple them with FM Ni thin-film overlayers. Using the symmetry-enabled piezostrains, we demonstrate robust 90° rotation of in-plane magnetic anisotropy in the Ni through application of just 3 V applied bias across the PMN-PT, compared to the >100 V required when using bulk single crystals of PMN-PT (*4*). We find that the piezostrains exhibited by the PMN-PT membrane can be attributed to driving the PMN-PT film’s polarization toward the O state under the applied bias, which, similar to bulk behavior, results in a nonpermanent effect. However, contrary to bulk behavior, we do not see evidence of the film switching into a metastable R_IP_ polarization state. To further understand the domain behavior of the PMN-PT membrane, we used high-resolution scanning transmission electron microscopy (STEM) to map B-site cation displacements to observe the domain configuration of the PMN-PT membrane. The STEM imaging shows that the membranes have a mixed relaxor and FE domain structure consisting of both in-plane and out-of-plane R polarizations, as well as domains with polarizations along lower symmetry directions, alluding to the presence of either orthorhombic (O) or monoclinic (M) phases. To aid in understanding the microstructural evolution under electric fields, phase-field simulations on the PMN-PT membrane were performed. The simulations demonstrate that the strain behavior of the PMN-PT membrane can be split into two regions, a low-field region where competing mechanisms result in relatively constant in-plane strain anisotropy as the macroscopic polarization of the membrane switches between up and down and a high-field region where polarization rotation between R_UP_ and O_UP_ dominates the strain behavior. This work furthers our understanding of the microscopic nature of relaxor-ferroelectric thin films, presenting a crucial step toward their use in low-power piezo-driven ME devices.

## RESULTS

### Fabrication of (011)-oriented membrane heterostructures

**Fig. 2.**
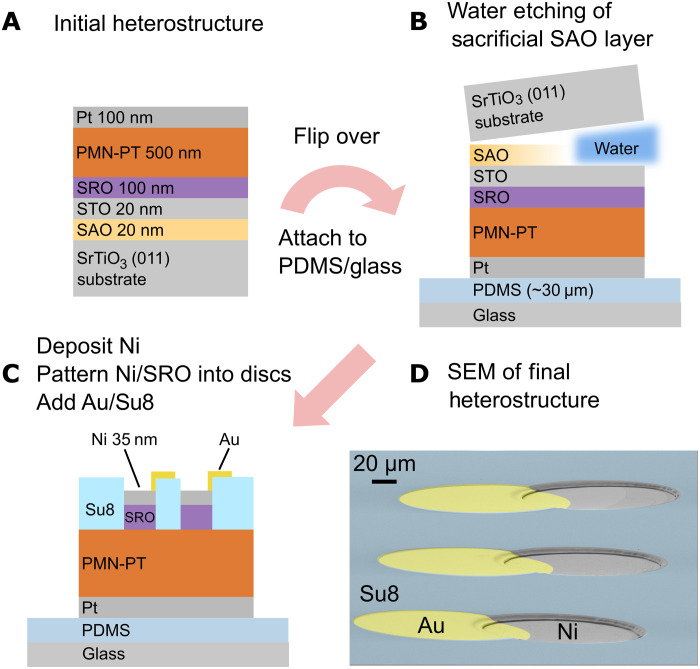
Fabrication of single-crystal (011)-oriented PMN-PT membrane heterostructures. (**A**) Initial thin-film heterostructure consisting of PLD-grown SAO/STO layers and sputter-deposited SRO/PMN-PT/Pt layers. (**B**) After attaching the heterostructure Pt-side into PDMS/Glass, the SAO sacrificial layer is etched by H_2_O. (**C**) After removal of the STO buffer layer, Ni is deposited by sputtering followed by patterning of the Ni/SRO layers into 160-μm circles. The membrane heterostructure is completed by addition of the SU-8 protective layer and Au-lifted electrode layer. (**D**) SEM image showing the completed membrane device.

Previously, we demonstrated the fabrication of (001)-oriented PMN-PT films on Si (*25*) and fabrication of membranes via etching of a Si substrate (*33*). Because of difficulties associated with growth of epitaxial PMN-PT on (011) Si, that method was incompatible for this study. Therefore, we instead use a (011)-oriented SrTiO_3_ (STO) substrate with a water-soluble Sr_3_Al_2_O_6_ (SAO) sacrificial layer to create the (011) membrane (*34*). Fabrication details can be found in Methods, and key steps are highlighted in Fig. 2. First, epitaxial SAO and a capping STO layer are grown on top of (011)-oriented STO substrates by pulsed laser deposition (PLD), followed by sputtering of epitaxial SrRuO_3_ (SRO) and PMN-PT layers (Fig. 2A). After depositing a Pt electrode, the entire heterostructure is then attached topside down onto a polydimethylsiloxane (PDMS) and glass platform followed by H_2_O etching of the SAO layer to release the films from the STO substrate (Fig. 2B). After removing the STO buffer layer, deposition and patterning of the FM Ni layer into 160-μm-diameter circular patterns, as well as deposition and patterning of protective polymer SU-8 and Au-lifted electrode top layers, result in the final membrane heterostructure shown in Fig. 2C. A scanning electron microscope (SEM) image of the final heterostructure is shown in Fig. 2D. To confirm that the PMN-PT retained its high-quality single-crystalline structure, x-ray diffraction was performed before and after substrate removal (fig. S1A). The PMN-PT out-of-plane lattice parameter exhibits no change upon removal of the substrate (fig. S1B), and the full width at half maximum of the (011) PMN-PT peak rocking curve remains the same as well (fig. S1C).

### Symmetry-enabled rotation of Ni in-plane anisotropy

**Fig. 3.**
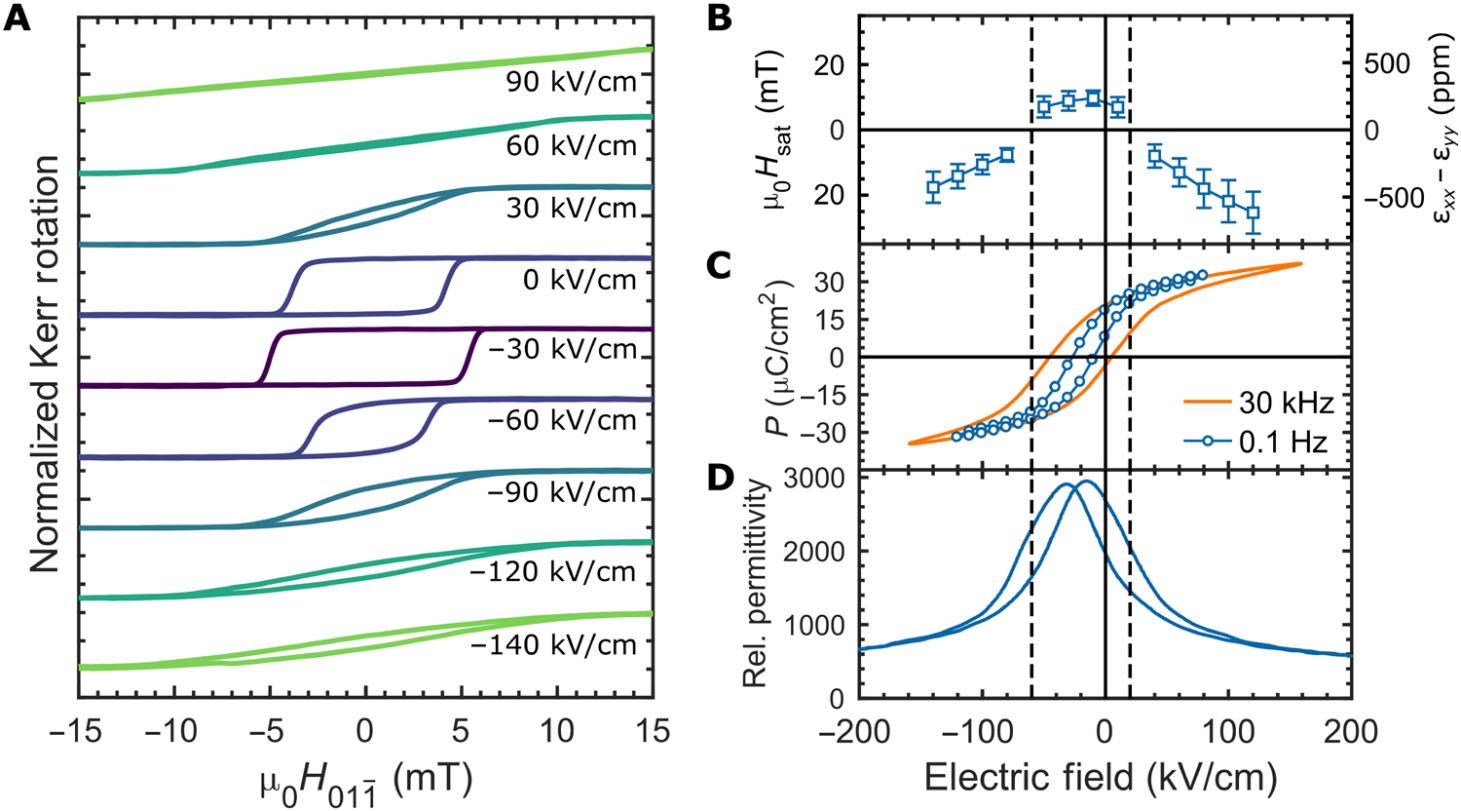
ME, FE, and piezoelectric properties of PMN-PT membrane heterostructures. (**A**) MOKE magnetic hysteresis loops (normalized) at a series of electric fields from −140 kV/cm (−7 V) to 90 kV/cm (4.5 V). Dark colors are closer to the FE imprint, and lighter colors are further from the imprint. (**B**) Saturation magnetic field (*H*_sat_; left axis) and calculated anisotropic strain (ε*_xx_* − ε*_yy_*; right axis) versus biasing electric field extracted from HA MOKE hysteresis loops similar to those shown at high-bias electric field in (A). Error bars represent the SD of measurements of seven different devices on the same membrane. Negative differential strain points (ε*_xx_* − ε*_yy_* < 0) were extracted from HA MOKE loops with magnetic field along$\left[01\bar{1}\right]$ and positive points (ε*_xx_* − ε*_yy_* > 0) from loops where magnetic field was along [100]. (**C**) Polarization (*P*) vs electric field hysteresis loop measurements using the 160-μm-diameter Ni/SRO top electrode. The orange loop was measured with a 30-kHz sinusoidal voltage pulse. The blue curve, labeled as 0.1 Hz, was acquired using a quasi-DC measurement procedure (see Methods). (**D**) Relative permittivity versus biasing electric field. Bias electric field was swept at 0.5 Hz, and permittivity was measured with a small AC electric field of 3.5 kV/cm RMS at 4 kHz. For (B) to (D), guidelines are added to separate the behavior into a low-field region (near FE imprint) and high-field regions.

Strain-induced changes of magnetic anisotropy in the Ni overlayer were measured by longitudinal magneto-optic Kerr effect (MOKE) hysteresis loops as a function of PMN-PT bias electric fields. When the applied magnetic field is swept along a magnetic easy axis (EA), a square hysteresis loop results from the magnetization reorienting between parallel and antiparallel to the applied field. A magnetic field applied along a hard axis (HA) continuously rotates the magnetization away from the EA, resulting in a linear hysteresis loop that saturates at full rotation. Therefore, with the field applied along the ${\left[01\bar{1}\right]}_{\text{pc}}$ *y* direction, we can observe a 90° rotation of magnetic anisotropy as the MOKE loop transitions from an EA to HA upon application of the electrical bias, as seen in Fig. 3A.

Because of Ni’s negative magnetostriction, it will align its EA along the more compressive direction in the presence of anisotropic strain. At 0 kV/cm bias, the as-grown Ni has a weak EA anisotropy along the *y* direction. Application of a large electric bias (positive or negative) results in HA MOKE loops (Fig. 3A, green curves), meaning that the strain is more compressive along the [100]_pc_ *x* direction. To confirm that the EA anisotropy is now along the *x* direction, MOKE loops were measured with the magnetic field parallel to the [100]_pc_ direction, rotated 90° in-plane from Fig. 3A (fig. S2), and EA MOKE loops were observed at high fields. This matches the expected strain behavior associated with driving the film toward O_UP_ symmetry (Fig. 1). When the electric bias is removed, however, the Ni returns to the as-grown state regardless of bias history, indicating that the strain is relaxed upon removal of the bias. Application of −30 kV/cm bias results in an EA MOKE loop with a higher coercive field (Fig. 3A, purple curve). The reinforcement of the EA along the *y* direction indicates a reversal in the strain anisotropy from the high-field case, i.e., more compressive along the ${\left[01\bar{1}\right]}_{\text{pc}}$ *y* direction, and fig. S2 confirms that the MOKE loop around the FE imprint shows a HA along the *x* direction. Therefore, we observe a 90° rotation of the Ni in-plane anisotropy over the range of −30 to 30 kV/cm applied bias, corresponding to a 3-V bias across the thickness of our 500-nm PMN-PT membranes. Overall, the MOKE hysteresis behavior is symmetric about −30 kV/cm, which we will show to be due to the FE imprint (discussed in the next section). Similar experiments were performed on a 500-nm clamped PMN-30PT thin film still attached to its STO substrate (fig. S3). Even up to an applied bias of ±400 kV/cm (±20 V), there is no change in the MOKE loop hysteresis. This demonstrates the importance of removing mechanical clamping by the substrate, without which the large anisotropic in-plane strains cannot be achieved.

### Strain behavior and FE properties of PMN-PT/Ni membranes

To understand the strain behavior inferred from the MOKE hysteresis, we plotted the calculated magnetic anisotropy energy density (*K*_U_) determined from the saturation field of HA loops and the associated differential strain (ε*_xx_* − ε*_yy_*) using the known magnetostriction of Ni in Fig. 3B. Polarization (*P*) versus electric field hysteresis loops (PE loops) are in Fig. 3C, and permittivity versus electric field are in Fig. 3D. Note S2 details the calculation of *K*_U_ and ε*_xx_* − ε*_yy_*. The PE loops in Fig. 3C show an FE imprint of approximately −30 kV/cm, which we believe to be due to the asymmetric electrode configuration of SRO (top) and Pt (bottom) (*35*). This results in the zero bias polarization of the PMN-PT film to be in a partially polarized state of ~15 μC/cm^2^ pointing toward the SRO electrode. The MOKE hysteresis loops (Fig. 3A), as well as the calculated strain (Fig. 3B) and permittivity (Fig. 3D), show similar symmetric behavior about the FE imprint.

In Fig. 3 (B to D), guidelines have been added that separate the membrane behaviors into three regions: a low-field region near the FE imprint and high-field regions away from the imprint. In the low-field region, we observe that the strain remains relatively constant (Fig. 3B), while the polarization is switching between negative (down) and positive (up) (Fig. 3C). As will be shown in the next section, the film at 0 kV/cm exhibits a mixture of both in-plane rhombohedral (R_IP_) and out-of-plane rhombohedral (R_OP_) domains. Within the low-field region, the film maintains this mixed R state, while the polarization switches between positive and negative, resulting in only minor changes to the differential strain. When the polarization begins to saturate in the high-field region, the strain exhibits the largest changes with applied bias as observed in Fig. 3B as well as the MOKE hysteresis in Fig. 3A. The high-field strain behavior arises from monoclinic distortions as the spontaneous polarizations of R_OP_ domains rotate toward the O direction, as demonstrated later in the “Phase-field simulations” section.

Another interesting feature of the PMN-PT membranes is that the PE loops show a slim-loop hysteresis with low remnant polarization. Similar PE behavior has been reported in other studies of PMN-PT thin films with similar composition (*15*, *16*, *25*, *36*) and resembles the PE hysteresis of a canonical relaxor becoming nonergodic, such as PMN (PMN-*x*PT with *x* = 0%) around 250 K (*37*). This supports the claim that the morphotropic phase boundary may be shifted to a higher PT content in PMN-PT thin films (*36*), suggesting that changing the composition of the film may increase the hysteresis of the membrane. The hysteresis also decreases as we approach the DC limit as seen by the quasi-static PE loop at 0.1 Hz (see Methods). Because all MOKE measurements must be performed under DC bias, the reduced hysteresis of the quasi-static loop demonstrates that the nonpermanent strain behavior observed in MOKE is closely related to the nonpermanent polarization behavior of the PMN-PT film.

In the study by Wu *et al.* (*4*) using bulk (011) PMN-PT with a Ni overlayer, as well as the study of nanosized Ni ellipses on bulk (011) PMN-PT by Buzzi *et al.* (*6*), the primary mechanism for anisotropy rotation in the Ni was anisotropic in-plane strain generated by permanent 71°/109° switching between R_IP_ and R_UP_ polarization states. A primarily R_IP_ state would be expected, where the overall polarization in the film approaches 0 μC/cm^2^. This would occur near the FE imprint (−30 kV/cm) as seen in Fig. 3C. As we will show in the next section, at 0 kV/cm, where the polarization is ~15 μC/cm^2^, we have a mixture of R_IP_ and R_OP_ domains. Therefore, transitioning from the mixed state at 0 kV/cm to a full R_IP_ state at −30 kV/cm would result in ε*_xx_* − ε*_yy_* < 0 (Fig. 1) and should result in a HA MOKE loop. However, Fig. 3A shows an EA MOKE loop at the imprint field instead. Therefore, we do not observe permanent switching to a fully R_IP_ state as observed in bulk (011) PMN-PT.

### STEM analysis of the PMN-PT membrane structure

**Fig. 4.**
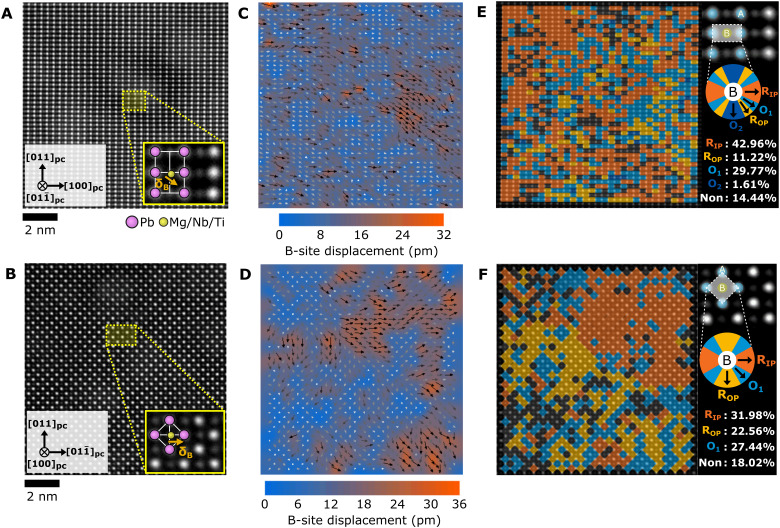
STEM analysis of domains present in the PMN-PT membrane. (**A** and **B**) Atomic resolution high-angle annular dark-field (HAADF)–STEM images along the${\left[01\bar{1}\right]}_{\text{pc}}$and [100]_pc_ zone axes, respectively. The insets are enlarged images in each zone axis. Pink circles are A-site cations (Pb) and yellow circles are B-site cations (Mg/Nb/Ti). Orange arrows are the B-site displacement (δ_B_). (**C** and **D**) B-site cation displacement mapping with overlaid arrows indicating regions of short-range ordering. Color maps show the atomic displacement magnitude, and arrows display the direction of atomic displacement. (**E** and **F**) Phase fraction mapping in each unit cell with color wheel by expected B-site displacement directions for R_IP_ (R_1_), R_OP_ (R_2_), and regions that have displacements between the R states labeled as orthorhombic O_1_ and O_2_. Color blank regions (Non) indicate the nonpolar region under the 7 pm of B-site displacement.

The domain structure of the as-grown PMN-PT membranes was investigated via STEM. Details of the STEM sample and analysis are in Methods. A cross-sectional image of the heterostructure is shown in fig. S4A. The PMN-PT shows a columnar structure with threading dislocations between the columns that arise owing to the lattice mismatch between PMN-PT and SRO/STO during the growth of the PMN-PT film. The selected area diffraction patterns of a single column on each zone axis (fig. S4, B and C) shows that the PMN-PT is single crystalline. Figure 4 (A and B) shows the atomic resolution imaging of the PMN-PT film along the ${\left[01\bar{1}\right]}_{\text{pc}}$ and [100]_pc_ zone axes, respectively. To observe the PMN-PT domain structure, we mapped the B-site cation displacement direction and magnitude using the atomic resolution images for both zone axes (Fig. 4, C and D). Another representation of the displacements is shown in Fig. 4 (E and F), where the regions are grouped by corresponding directions for both R and O polarizations for each zone axis (see the color wheel next to the images).

Because the STEM is performed at zero bias, the FE imprint causes many of the B-site cations to displace either in-plane or down toward the Pt electrode. As seen in Fig. 4 (C to F), regions of correlated displacements range from less than 1 nm up to a few nanometers in size. These nanoscale domains vary in direction with smooth transitions between them, consisting of both R_IP_ (R_1_) and R_OP_ (R_2_) displacements, as well as displacement directions in between that cannot be classified as either R direction. We label these regions as an orthorhombic O_1_ and O_2_, but they could correspond with monoclinic distorted unit cells with polarizations that lie between the two R states as well. Because of the presence of both R_IP_, R_OP_, and polarization states in between them, we expect the overall differential in-plane strain state at 0 kV/cm to be somewhere between the R_UP_ and R_IP_ states, likely close to the zero strain cubic reference state in Fig. 1C. These findings closely resemble the cation displacement measurements from TEM performed by Kumar *et al.* (*38*) and resemble a mixed FE and relaxor domain structure consistent with the polar slush model (*39*).

### Phase-field simulations of PMN-PT membrane

**Fig. 5.**
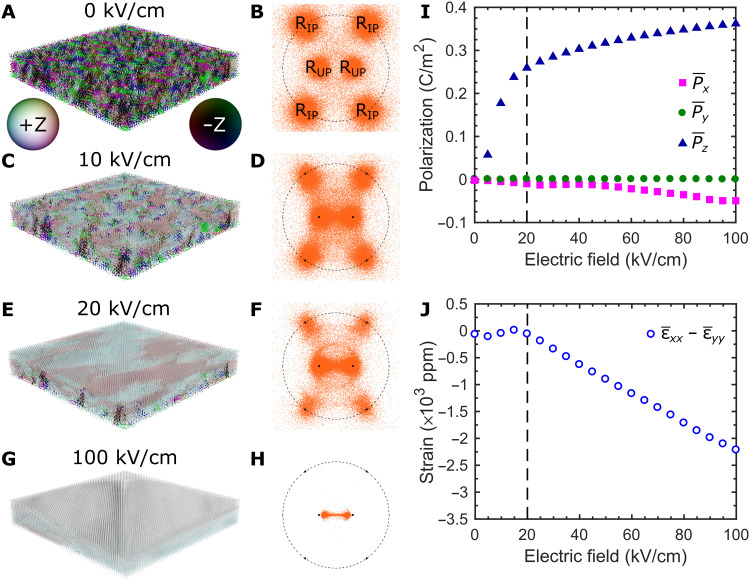
Phase-field simulations of the (011) PMN-PT membrane. Spontaneous polarization and [011] stereographic projection of the PMN-PT membrane at (**A** and **B**) 0 kV/cm, (**C** and **D**) 10 kV/cm, (**E** and **F**) 20 kV/cm, and (**G** and **H**) 100 kV/cm. The legend for the coloring of spontaneous polarization is included in (A). (**I**) Average polarization in the *x*, *y*, and *z* directions versus applied field. (**J**) Field dependence of the average anisotropic in-plane strain${\bar{\varepsilon }}_{\mathrm{xx}}-{\bar{\varepsilon }}_{\mathrm{yy}}$. In (I) and (J), guidelines have been added to separate the low-field and high-field regions.

Phase-field simulations were performed to understand the strain behavior of the PMN-PT membrane. Details of the simulation can be found in Methods. The initial domain configuration consists of a mixture of R_IP_ and R_OP_ as seen in the spontaneous polarization diagram in Fig. 5A, as well as the [011] (*z* direction) stereographic projection of the spontaneous polarizations in Fig. 5B. The evolution of the spontaneous polarization distribution with electric field is shown for 10 kV/cm (Fig. 5, C and D), 20 kV/cm (Fig. 5, E and F), and 100 kV/cm (Fig. 5, G and H). The simulation is summarized by plotting the average polarization for the *x*, *y*, and *z* directions (Fig. 5I), as well as the average in-plane strain (Fig. 5J). The average strain was calculated by averaging the strain contribution of individual spontaneous polarization elements multiplied by the electrostriction tensor described in note S1. In the simulation, 0 kV/cm corresponds to no electric bias across the PMN-PT membrane, including any built-in bias from an FE imprint. Therefore, the starting point of the simulation corresponds to the expected structure around the FE imprint of the experimental PMN-PT membrane (−30 kV/cm). In addition, histograms of the absolute angle between spontaneous polarizations and the O_UP_ [011] direction are shown in fig. S5.

Guidelines are added to Fig. 5 (I and J) to separate low-field behavior from high-field behavior. As seen in Fig. 5I, the polarization increases rapidly in the low-field region as the mixed R_IP_, R_DOWN_, and R_UP_ state switches to primarily R_UP_ by 20 kV/cm (Fig. 5, C to F, and fig. S5, B and C). Within this region, we observe two behaviors of the spontaneous polarizations within the PMN-PT: (i) polarization switching from R_DOWN_/R_IP_ to R_UP_, as seen in the decrease in polarizations near the R_IP_ regions of the stereographic projection, and (ii) polarization rotation from R_UP_ to O_UP_, as indicated by the region between the two R_UP_ polarizations being populated in the stereographic projections. The increase in R_UP_ polarizations and polarization rotation toward O_UP_ (indicated by the shift in the peak toward a lower angle) are also visible in fig. S5. While the switching between R_DOWN_ and R_UP_ does not result in a change of in-plane strain, the switching between R_IP_ to R_UP_ results in ε*_xx_* − ε*_yy_* > 0 (Fig. 1C). On the other hand, the polarization rotation between R_UP_ and O_UP_ results in ε*_xx_* − ε*_yy_* < 0. Therefore, the low-field region experiences a large increase in average polarization in the *z* direction, while the competing strain behaviors act to keep the in-plane strain relatively constant (Fig. 5, I and J).

In the high-field region, the average polarization is nearly saturated but continues to increase as the polarization continues to rotate from R_UP_ toward the O_UP_ state. Nearly all of the polarizations have been switched to R_UP_, meaning that the polarization rotation toward O_UP_ dominates the strain behavior resulting in a large decrease in differential in-plane strain (Fig. 5J). By 100 kV/cm, all of the polarizations lie in the region between R_UP_ and O_UP_ (Fig. 5, G and H, and fig. S5D), corresponding to a large monoclinic distortion away from R_UP_. The simulation results qualitatively agree with the experimental strain and polarizations measured in the PMN-PT/Ni membrane (Fig. 3), showing relatively constant in-plane strain during polarization switching and large changes of in-plane strain at higher fields. Note that the strains calculated from the experimental MOKE loops (Fig. 3B) exhibit a horizontal and vertical shift relative to the calculated strains from simulation (Fig. 5J), although, qualitatively, the two curves are similar. The vertical shift arises from the as-grown Ni being weakly anisotropic with the EA along the *y* direction at 0 kV/cm, while the horizontal shift comes from the FE imprint of the PMN-PT membranes being approximately −30 kV/cm.

## DISCUSSION

We have provided demonstration of the low-voltage strain-mediated ME effect in an all-thin-film heterostructure that only relies on the large anisotropic strains inherent to (011) PMN-PT thin films by completely removing them from their substrate. The PMN-PT/Ni membranes achieve a robust, piezo-driven, 90° rotation of the in-plane magnetic anisotropy of the Ni overlayer under application of only a few volts of bias across the thickness of the PMN-PT membrane. This is roughly two orders of magnitude less voltage than demonstrations using bulk single crystals of PMN-PT that require application of >100 V (*4*). The ME coupling is achieved by driving the PMN-PT polarization toward orthorhombic symmetry under the applied bias, resulting in strain anisotropy controlled by the in-plane crystal symmetry of the PMN-PT film. STEM measurements show that the zero-field domain structure of the PMN-PT membrane consists of a mixture of both R_IP_ and R_OP_ domains, as well as additional regions with B-site cation displacements along directions between the two R states. Phase-field simulations confirm that the in-plane differential strain does not change in the low-field region near the FE imprint because of the switching between R_IP_ and R_OP_ domains competing with polarization rotation toward O_Up_. However, at higher fields, polarization rotation toward O_Up_ dominates and once again results in a giant piezoelectric effect.

Demonstrations using bulk PMN-PT show permanent switching between in-plane and out-of-plane R polarization states and, consequently, between distinct strain states. The permanent switching behavior of bulk PMN-PT is typically a trait that is deemed desirable for applications such as memory storage, but the nonpermanent strains in our PMN-PT membranes may still be able to provide the 180 magnetization switching needed for memory devices (*40*, *41*). Understanding the differences between bulk and membrane PMN-PT response to external stimuli is key to their use in future technologies. Such differences may arise owing to various effects, such as smaller domain sizes in membrane versus bulk, reduced chemical ordering in PMN-PT membranes due to differences in material processing parameters (e.g., growth temperature), as well as different electrical/mechanical boundary conditions in membranes arising from higher defect concentration, or enhanced role of the interface (*42*). Studying the membrane PMN-PT electrical bias response using in situ STEM or synchrotron x-ray diffraction, as well as studying compositions other than the *x* = 30% (1 − *x*)PMN-(*x*)PT used here, could provide key information for future applications. Our work provides key insight into the microstructural behavior of PMN-PT thin-film membranes and demonstrates how they can be used in ME coupling devices. In addition, coupling the PMN-PT membrane with a variety of other materials, such as complex oxides, two-dimensional materials, and III-V semiconductors, can lead to the discovery of previously unknown piezo-driven phenomena.

## METHODS

### Membrane fabrication

Twenty nanometers of epitaxial SAO was grown on top of (011) STO substrates via PLD. The SAO was grown at a substrate temperature of 780°C and *p*(O_2_) = 1 × 10^−6^ torr using a laser fluence of 1 J/cm^2^ on the polycrystalline SAO target. A 20-nm capping layer of STO, preventing possible cation diffusion at the interface (*43*), was grown at 750°C and *p*(O_2_) = 1 × 10^−6^ torr using a laser fluence of 2 J/cm^2^ on the single-crystal STO target. One hundred nanometers of SRO was grown by radio frequency (RF) magnetron sputtering at a power of 100 W in 200 mtorr of Ar:O_2_ (12:8) with a substrate temperature of 600°C. Five hundred nanometers of 70% Pb(Mg_1/3_Nb_2/3_)O_3_–30% PbTiO_3_ (PMN-30PT) was grown by RF magnetron sputtering at a power of 100 W in 500 mtorr of Ar:O_2_ (17:3) with a substrate temperature of 625°C. One hundred nanometers of Pt was deposited at room temperature by DC magnetron sputtering. The edges of the heterostructure were ground slightly to remove any sidewall deposition of SRO, PMN-PT, or Pt that would prevent H_2_O from etching the SAO later on.

PDMS with a weight ratio of 10:1 (base:cross-linking agent) was spin-coated onto a 10 mm by 10 mm glass substrate at 5000 rpm for 10 s, resulting in a PDMS thickness of ~30 μm. Before the PDMS is cured, the PMN-PT film heterostructure is placed into the PDMS Pt-side down. The entire sample is placed in vacuum for a minimum of 5 hours to remove any bubbles between the PDMS and Pt layers, followed by curing of the PDMS on a hot plate at 100°C for 1 hour. Placing the film into the uncured PDMS is crucial, as it allows the PDMS to mold to the surface of the film, ensuring that the PMN-PT membrane is as flat as possible after the substrate is removed. One consequence of curing the PDMS after the film is attached is that it will mold to the sides of the substrate. Once cured, removing the PDMS from the sides of the substrate with a razor is necessary so that the H_2_O can reach the SAO.

The sample is placed in a beaker of water to etch the sacrificial SAO layer. This process can take anywhere from 24 to 72 hours. Heating the water to 70° to 80°C was found to speed up the etching in some cases. Etching progress was monitored by visual inspection under a microscope, and once found to be completed, the substrate was removed using tweezers. Dipping in isopropyl alcohol was used to displace water and reduce surface tension between the substrate and film in some instances.

With the substrate removed, the exposed surface of the membrane now consists of the STO layer that was used to cap the SAO layer. Ion milling was used to remove the STO layer and expose the SRO film. A 35-nm Ni film was deposited by DC sputtering at room temperature on top of the SRO to act as the FM layer for our FE/FM composite. Photolithography and wet etching were used to pattern the SRO/Ni into 160-μm-diameter disks. SU-8 photoresist was spin-coated and patterned by photolithography to create a protection layer that left the Ni/SRO disks exposed while covering the PMN-PT. Thirty nanometers of Au was deposited by DC sputtering at room temperature, followed by photolithography and patterning to create Au electrodes partially overlapping the Ni/SRO disks, and partially on top of the SU-8. This allowed for electrical contact to be made with probe tips or wire bonding to the Au on the SU-8 layer without risk of damaging the fragile membrane heterostructure.

### Scanning transmission electron microscopy

Two kinds of cross-sectional samples having [100] and $\left[01\bar{1}\right]$ pseudo-cubic projections were prepared using a dual-beam focused ion beam system (Helios G3, FEI) to determine the FE domain structure. We used a Ga ion beam at 30 kV to make a thin specimen and then used different acceleration voltages from 5 to 1 kV for the sample cleaning process to reduce the Ga damage. The selected area diffraction pattern analysis and atomic structure observation were performed using a STEM (JEM-ARM200F, JEOL, Japan) at 200 kV equipped with a fifth-order probe corrector (ASCOR, CEOS GmbH, Germany) at the Materials Imaging and Analysis Center of Pohang University of Science and Technology (POSTECH) in South Korea. The optimum size of the electron probe for STEM observation was ~78 pm. The collection semiangles of the high-angle annular dark-field (HAADF) detector were adjusted from 68 to 280 mrad to collect scattered electrons in a large angle for clear *Z*-sensitive images. HAADF-STEM images were acquired using SmartAlign (HREM Research Inc., Japan), which conducted the multistack of images and aligned them using rigid registration to correct the sample drift and scan distortions. The obtained raw images were processed using a band-pass difference filter with a local window to reduce background noise (Filters Pro, HREM Research Inc., Japan).

STEM image analysis was performed by MATLAB with the customized atomic analysis code. All atomic coordinates were determined by the centroid of each atomic column. Chemical classification on A and B site was conducted with *Z*-contrast difference on the HAADF-STEM image, and unit cell was defined as B site with four neighboring A sites in [100]_pc_ projection and B site with six neighboring A sites in ${\left[01\bar{1}\right]}_{\text{pc}}$ projection. B-site off centering was defined by the displacement of B-site position from the mean neighbor positions of A sites for each unit cell.

### Phase-field simulations

The phase-field method is used to simulate the effect of applied bias on the polarization distribution in (011) PMN-30PT freestanding membranes. In the phase-field model, the polarization is selected as the order parameter to describe the domain structures, and its spatial and temporal evolutions are controlled by the time-dependent Ginzburg-Landau equation (*44*, *45*): (*∂P*_i_/*∂t*) = −*L*(δ*F*/δ*P*_i_), with *L* being the kinetic coefficient related to the domain-wall mobility and *F* being the total free energy, which includes the bulk chemical energy, polarization gradient energy, electric energy, and elastic energy (*44*). The parameters for the bulk chemical energy are from literature (*46*). The freestanding membrane is represented by a grid of 128Δ*x* × 128Δ*x* × (*N*_bottom-air_ + *N*_membrane_ *+ N*_top-air_)Δ*z* with Δ*x* = Δ*z =* 1 nm, *N*_bottom-air_ = 2, *N*_membrane_ *=* 20, and *N*_top-air_ = 2. Periodic boundary conditions are assumed in the in-plane directions. To consider the (011) orientation, we define the simulation coordinate system (*x*, *y*, *z*) to be *x*//[100], *y*//$\left[01\bar{1}\right]$, and *z*//[011], and the tensor coefficients are rotated by the corresponding rotation matrix.

Stress-free boundary conditions are assumed for both the top and bottom surfaces for solving the elastic equilibrium equation of the membrane. This is achieved by considering an inhomogeneous system including two layers of vacuum at both the top and bottom surfaces of the membrane. The elastic equilibrium equation of such an inhomogeneous system is solved using the spectral iterative perturbation method (*47*). To incorporate the applied electric field, a superposition method is used to solve the electrostatic equilibrium equation with a uniform preset voltage bias at the top surface while the bottom surface is grounded (*48*).

### MOKE measurements

The PMN-PT membrane was mounted between the poles of an electromagnet and a polarized red HeNe (632 nm) laser was reflected off of the sample surface at approximately 45° from normal incidence. The beam was focused to an approximately 10-μm spot near the center of the 160-μm Ni discs using an achromatic lens. The reflected beam’s polarization was analyzed with a differential detection scheme. A polarizing beam splitter directed the *s* and *p* components of the reflected beam onto two channels of a Thorlabs PDB210A differential photodetector and a half–wave plate before the beam splitter was used to balance the inputs to the detector. A fitting procedure was used to extract *H*_sat_ from HA MOKE loops, resulting in the values plotted in Fig. 4D. The loop was normalized so that the Kerr rotation at magnetic saturation is ±1. Data with normalized Kerr rotation values between 0.85 and −0.85 were fit to a line. The magnetic field values where the fitted line intersects with +1 and −1 normalized Kerr rotation were respectively taken to be $H_{\text{sat}}^{+}$ and $H_{\text{sat}}^{-}$, with *H*_sat_ as the average.

### FE measurements

Permittivity measurements were performed by slowly (0.5 Hz) sweeping a bias electric field across a device while also applying an AC waveform [3.5 kV/cm root mean square (rms) at 4 kHz], detecting the resulting 4-kHz AC current with a lock-in amplifier. High-frequency PE loops were obtained by integrating the current flowing to the device while sweeping the electric field at 30 kHz. The observed frequency dependence of the PE loop required determining the PE response at very low frequency to compare with MOKE measurements, where magnetic hysteresis data were acquired with static electric field. A 0.1-Hz equivalent polarization at an electric field *E*_0_ was determined by letting the polarization decay from its initial high-frequency sweep value for 10 s at fixed electric field *E*_0_ and then saturating with a high-frequency sweep and integrating the resulting current to determine the polarization change. Both the initializing sweep and saturating sweep were complete PE loops with the same electric field extent, but phase-shifted to begin and end at *E*_0_.

## Supplementary Materials

### This PDF file includes:

Figs. S1 to S5 Table S1 Notes S1 and S2 References
