# Supplementary material for: Low-voltage magnetoelectric coupling in membrane heterostructures
Source: Sci Adv. 2021 Nov 12;7(46):eabh2294. doi: 10.1126/sciadv.abh2294 (PMC8589311; doi:10.1126/sciadv.abh2294)
Supplement: Supplementary file 1 — Figs. S1 to S5 Table S1 Notes S1 and S2 References [file sciadv.abh2294_sm.pdf]

Science Advances
AAAS

Supplementary Materials for

Low-voltage magnetoelectric coupling in membrane heterostructures

Shane Lindemann, Julian Irwin, Gi-Yeop Kim, Bo Wang, Kitae Eom, Jianjun Wang, Jiamian Hu, Long-Qing Chen, Si-Young Choi, Chang-Beom Eom*, Mark S. Rzchowski*

*Corresponding author. Email: eom@engr.wisc.edu (C.B.E.); rzchowski@physics.wisc.edu (M.S.R.)

Published 12 November 2021, *Sci. Adv.* **7**, eabh2294 (2021)
DOI: 10.1126/sciadv.abh2294

This PDF file includes:

Figs. S1 to S5
Table S1
Notes S1 and S2
References

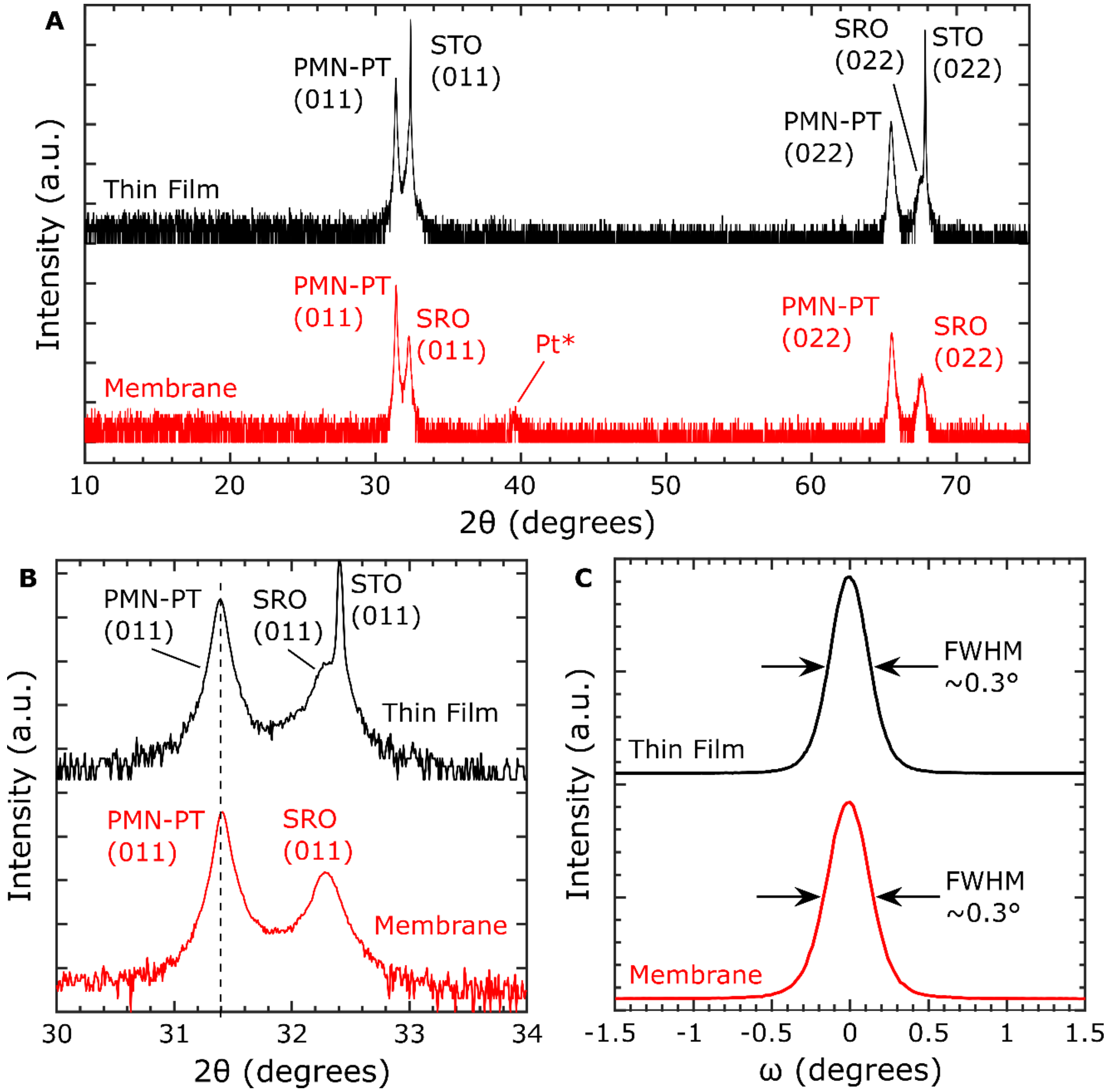

Fig. S1. XRD structural characterization of PMN-PT thin film before and after release from substrate. (A) θ-2θ scan showing that there are no secondary phases that appear in the PMN-PT thin film even after substrate removal. The SRO (011) peak is too close to the substrate to see in the film scan but appears after the substrate is released. There are no STO substrate peaks in the membrane scan due to the substrate having been completely removed. An additional Pt peak is visible in the PMN-PT membrane due to the deposition of Pt as the back electrode having been done before film release (see methods). **(B)** θ-2θ scan of the region around the (011) peaks. The SRO (011) peak is now visible in both the thin film and membrane scans. A vertical dashed line shows that the (011) PMN-PT peak does not shift much with position, indicating that upon release of the substrate the out-of-plane lattice parameter of the PMN-PT does not change significantly. **(C)** Phi scan of the PMN-PT (011) peaks in both before and after release look identical and have identical full width half maximums (FWHM), showing that the crystallinity of the film does not change after release from the substrate.

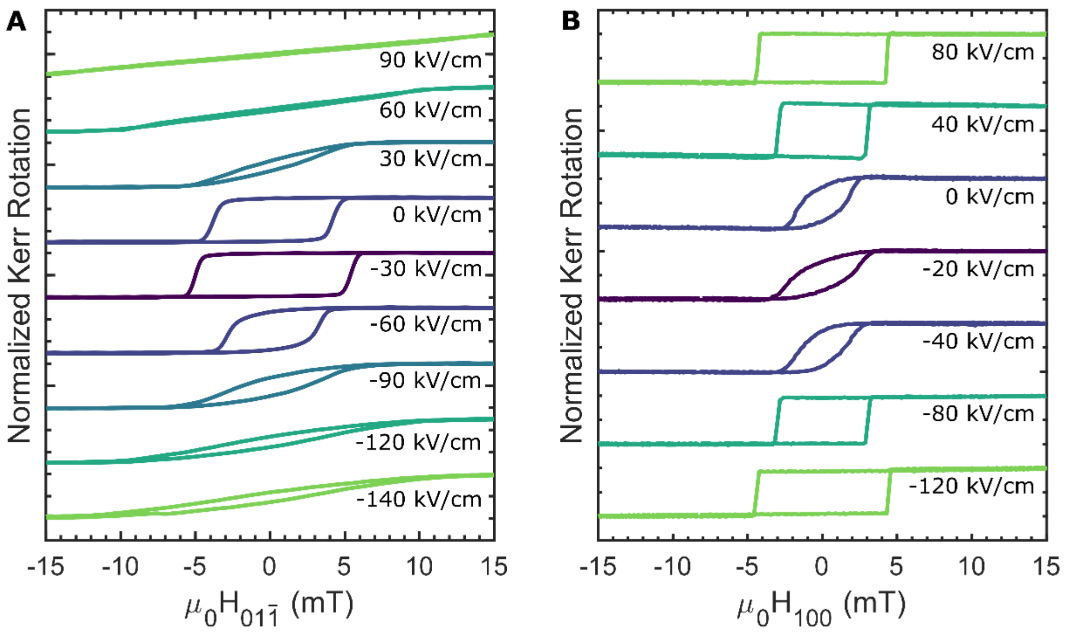

Fig. S2. MOKE hysteresis loops with applied magnetic field along both [$01\bar{1}$] and [100]. MOKE hysteresis loops taken on the PMN-PT/Ni membrane with the magnetic field applied along [$01\bar{1}$] y-direction (A, same as in Figure 3A), as well as 90 degrees rotated in-plane along the [100] x-direction. At high fields, the square MOKE loops in (B) show that the EA anisotropy of the Ni is aligned along the x-direction due to compressive strains caused by polarization rotation towards the O_{UP} direction (Figure 1). The slight tilting of the MOKE hysteresis at -20 kV/cm shows that the HA anisotropy is along the x-direction due to tensile strain along x.

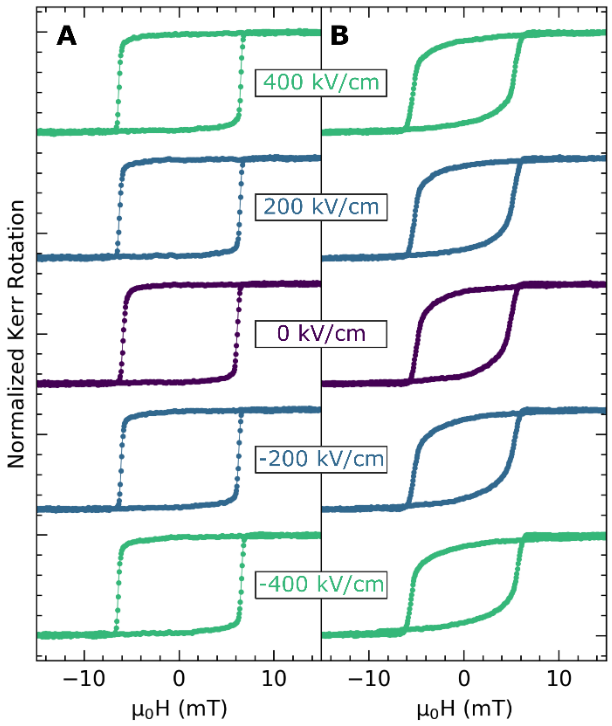

Fig. S3. MOKE hysteresis loops with applied electric field in clamped Ni/PMN-PT thin films. (A) Magnetic field aligned 30° from *x* [100] direction. **(B)** Magnetic field aligned 30° from the *y* $[01\bar{1}]$ direction. No electric field induced anisotropy was present in clamped thin film samples, even up to 400 kV/cm applied bias (corresponding to 20V in our thin films). Samples used were Ni (35 nm) / PMN-PT (500 nm) / $SrRuO_3$ (100 nm) / $SrTiO_3$ and all films were sputtered as described in Methods. Ni top electrodes were patterned into 300 μm by 200 μm rectangles. Hysteresis loops of same electric field magnitude have the same color.

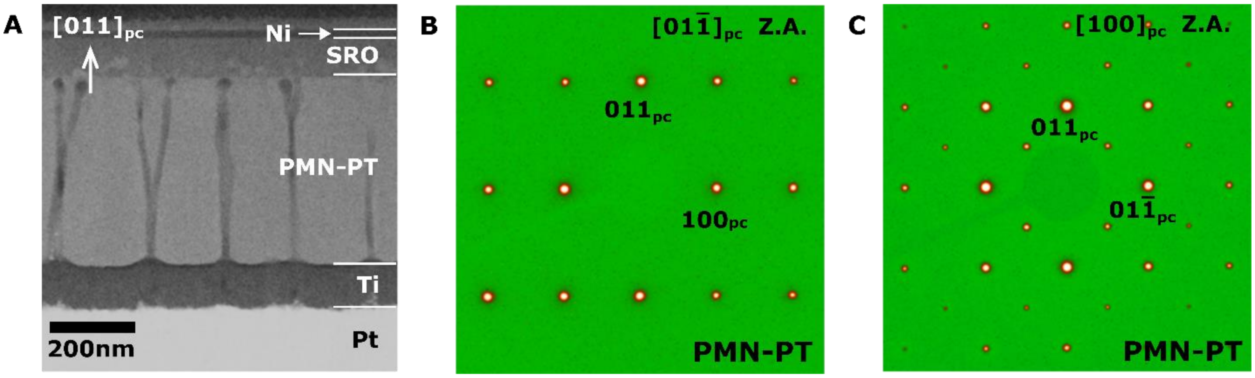

Fig. S4. Cross-sectional structure image and selected area diffraction patterns of PMN-PT membrane. (A) Cross section STEM image of the PMN-PT membrane heterostructure shows columnar structure. **(B-C)** Selected area diffractions of the PMN-PT membranes along the [01-1] and [100] pseudocubic zone axis show the membranes are single crystalline with a pseudocubic symmetry.

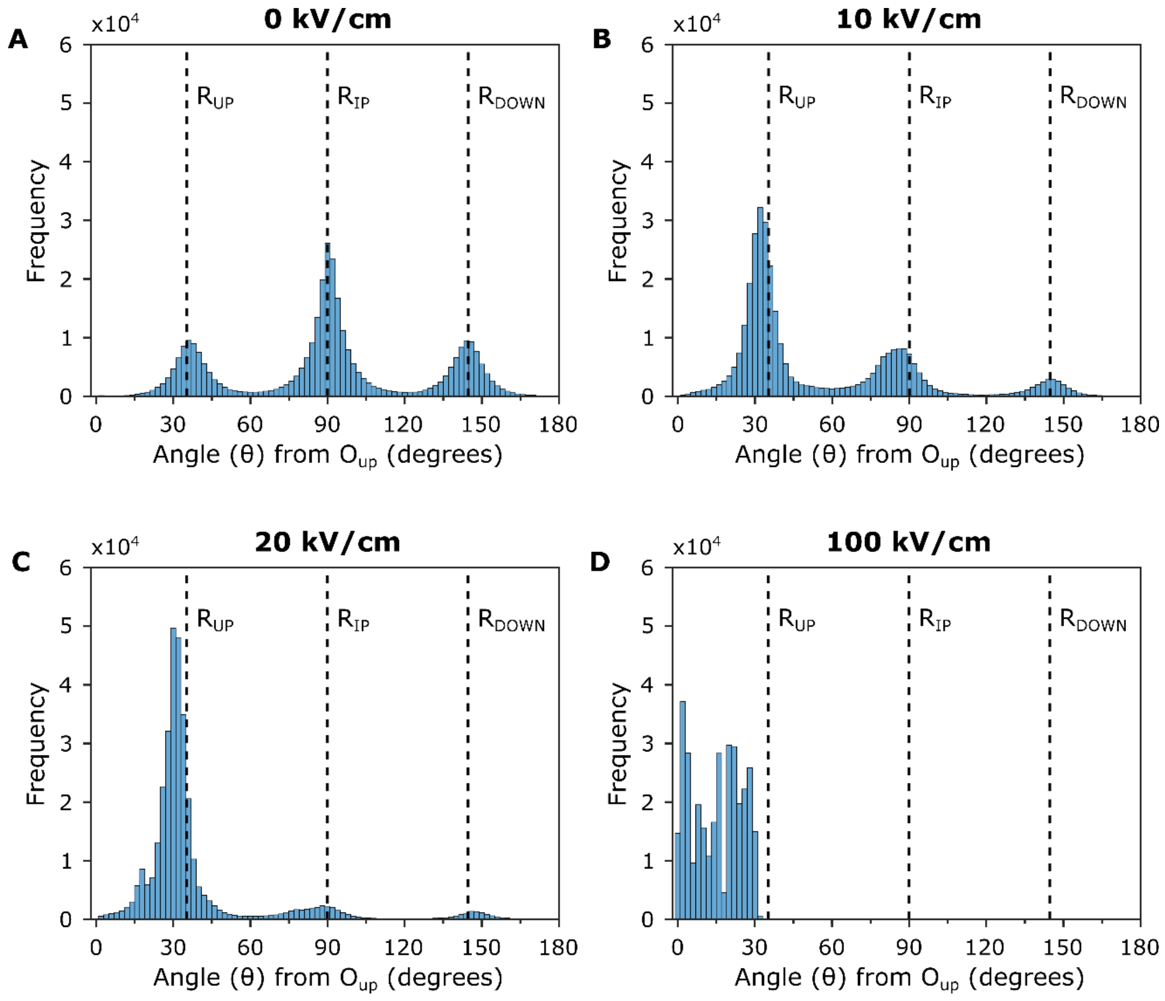

Fig. S5. Phase-field simulation polarization angle histograms. (A) At 0kV/cm, the simulation presents an even mixture of spontaneous polarizations along the R variants. **(B)** For a 10 kV/cm field applied along the z-direction [011], R_{IP} and R_{DOWN} domains switch to R_{UP} domains. **(C)** By 20 kV/cm, almost all polarizations are near the R_{UP} polarization direction, however, there is a shift towards a lower θ, indicating a monoclinic distortion towards the O_{UP} direction. **(D)** In the high field region, all of the spontaneous polarizations are in the monoclinic region between O_{UP} and R_{UP}, resulting in a large and negative differential in-plane strain. In each plot, the calculated angle for R_{UP}, R_{IP}, and R_{DOWN} polarizations, assuming a pseudocubic unit cell, are shown by dashed lines.

Supplementary Note S1 and Table S1: Calculated Polarization Group Strains

The electrostriction tensor is used to calculate the strain arising from an electric polarization. These strains are relative to the paraelectric cubic phase. The strain tensor ε_{ij} for a particular polarization vector P_k and electrostriction tensor Q_{ijkl} is

$$\varepsilon_{ij} = Q_{ijkl} P_k P_l \tag{1}$$

This equation may be written in the more compact Voigt matrix notation for cubic symmetry(*49*, *50*)

$$\begin{pmatrix} \varepsilon_{11} \\ \varepsilon_{22} \\ \varepsilon_{33} \\ 2\varepsilon_{23} \\ 2\varepsilon_{31} \\ 2\varepsilon_{12} \end{pmatrix} = \begin{pmatrix} Q_{1111} & Q_{1122} & Q_{1122} & 0 & 0 & 0 \\ Q_{1122} & Q_{1111} & Q_{1122} & 0 & 0 & 0 \\ Q_{1122} & Q_{1122} & Q_{1111} & 0 & 0 & 0 \\ 0 & 0 & 0 & 4\,Q_{1212} & 0 & 0 \\ 0 & 0 & 0 & 0 & 4\,Q_{1212} & 0 \\ 0 & 0 & 0 & 0 & 0 & 4\,Q_{1212} \end{pmatrix} \begin{pmatrix} P_1^2 \\ P_2^2 \\ P_3^2 \\ P_2 P_3 \\ P_1 P_3 \\ P_1 P_2 \end{pmatrix} \tag{2}$$

This can also be equivalently written with the commonly used matrix components Q_{ij}

$$\begin{pmatrix} \varepsilon_1 \\ \varepsilon_2 \\ \varepsilon_3 \\ \varepsilon_4 \\ \varepsilon_5 \\ \varepsilon_6 \end{pmatrix} = \begin{pmatrix} Q_{11} & Q_{12} & Q_{12} & 0 & 0 & 0 \\ Q_{12} & Q_{11} & Q_{12} & 0 & 0 & 0 \\ Q_{12} & Q_{12} & Q_{11} & 0 & 0 & 0 \\ 0 & 0 & 0 & Q_{44} & 0 & 0 \\ 0 & 0 & 0 & 0 & Q_{44} & 0 \\ 0 & 0 & 0 & 0 & 0 & Q_{44} \end{pmatrix} \begin{pmatrix} P_1^2 \\ P_2^2 \\ P_3^2 \\ P_2 P_3 \\ P_1 P_3 \\ P_1 P_2 \end{pmatrix} \tag{3}$$

where $\varepsilon_1 = \varepsilon_{11}$, $\varepsilon_2 = \varepsilon_{22}$, $\varepsilon_3 = \varepsilon_{33}$, $\varepsilon_4 = \varepsilon_{23}$, $\varepsilon_5 = \varepsilon_{31}$, $\varepsilon_6 = \varepsilon_{12}$, $Q_{11} = Q_{1111}$, $Q_{12} = Q_{1122}$, and $Q_{44} = 2\,Q_{1212}$.

The 1, 2 and 3 indices of Eq. (2) refer respectively to the cubic crystal directions [100], [010], and [001]. Values for Q_{ij} in Eq. (3) were taken from measurements reported in the literature on 28% rhombohedral PMN-PT crystals.(*12*) The strain tensor for a polarization group is found by equal weight averaging of the strain tensors calculated for each polarization vector present in the group.

The deformed unit cell for a particular polarization group is found from the principal strains of the strain tensor ε_{ij} (constructed from the ε_i and the symmetry condition $\varepsilon_{ij} = \varepsilon_{ji}$). The principal strains are three normal strains along directions given by the eigenvectors of ε_{ij} with magnitudes given by the eigenvalues of ε_{ij} . Each of the polarization group averages R_{Up}, R_{IP}, and O_{Up} result in principal strain directions aligned with the *x*, *y*, and *z* directions shown in Fig. 1A of the main text. The strain magnitudes along each direction, and the in-plane anisotropic strain $\varepsilon_{xx} - \varepsilon_{yy}$, are listed in Supplementary Table S1, both in terms of electrostriction matrix coefficients and numerically, with a 33 μC/cm^2 polarization magnitude. There is no shear strain since the strain

tensor is by definition diagonal in the *xyz* coordinate system defined by the strain tensor eigenvectors.

Supplementary Table S1 Electrostriction of polarization mixtures in (011)-oriented bulk PMN-PT. Strains were calculated analytically from $\varepsilon_{ij} = Q_{ijkl}P_kP_l$, and the 28% rhombohedral electrostriction tensor (Q_{ijkl}) values(*12*) were used to compute strains in ppm (shown in parentheses). Saturation polarization P was taken to be 33 μC/cm^2. The frequently occurring term $Q_h = Q_{11} + 2Q_{12}$ is the hydrostatic electrostriction coefficient.(*51*)

Strain	R_{IP}	R_{Up}	O_{Up}
ε_{xx}	$P^2\ \frac{1}{3}Q_h$ (254)	$P^2\ \frac{1}{3}Q_h$ (254)	$P^2\ Q_{12}$ (-2613)
ε_{yy}	$P^2\frac{1}{3}(Q_h + Q_{44})$ (1016)	$P^2\frac{1}{3}(Q_h - Q_{44})$ (-508)	$P^2\frac{1}{2}(Q_{11} + Q_{12} - Q_{44})$ (544)
ε_{zz}	$P^2\frac{1}{3}(Q_h - Q_{44})$ (-508)	$P^2\frac{1}{3}(Q_h + Q_{44})$ (1016)	$P^2\frac{1}{2}(Q_{11} + Q_{12} + Q_{44})$ (2831)
$\varepsilon_{xx} - \varepsilon_{yy}$	$-P^2\frac{1}{3}Q_{44}$ (-762)	$P^2\frac{1}{3}Q_{44}$ (762)	$P^2\frac{1}{2}(-Q_{11} + Q_{12} + Q_{44})$ (-3158)

Supplementary Note S2: Calculation of Ku and $\varepsilon_{xx} - \varepsilon_{yy}$

In the case of a hard axis loop, the magnetic anisotropy energy density K_U can be estimated using the experimental value of the full rotation field H_{sat} to be

$$K_U = \frac{1}{2}\mu_0 M_{sat} H_{sat} \tag{4}$$

where M_{sat} is the Ni saturation magnetization, assuming a coherent rotation model.(*52*) The strain induced anisotropy energy density is related to the Ni average anisotropic strain $\varepsilon_{xx} - \varepsilon_{yy}$ along principal strain axes by

$$K_U = \frac{3}{2}\lambda_s Y_{Ni}(\varepsilon_{xx} - \varepsilon_{yy}) \tag{5}$$

where λ_s is the Ni magnetostriction coefficient (-33 ppm) and Y_{Ni} is the Young's modulus of Ni (220 GPa).(*53*) Equations (4) and (5) allow the average anisotropic strain magnitude to be estimated from hard axis MOKE hysteresis loops. The sign of λ_s for Ni dictates that $\varepsilon_{xx} - \varepsilon_{yy} > 0$ results in a magnetic easy axis along $[01\bar{1}]$, and $\varepsilon_{xx} - \varepsilon_{yy} < 0$ results in an easy axis along $[100]$.

REFERENCES AND NOTES

1. J.-M. Hu, L.-Q. Chen, C.-W. Nan, Multiferroic heterostructures integrating ferroelectric and magnetic materials. *Adv. Mater.* **28**, 15–39 (2016).

2. Z. Chu, M. PourhosseiniAsl, S. Dong, Review of multi-layered magnetoelectric composite materials and devices applications. *J. Phys. D. Appl. Phys.* **51**, 243001 (2018).

3. S. Zhang, F. Li, High performance ferroelectric relaxor-$PbTiO_3$ single crystals: Status and perspective. *J. Appl. Phys.* **111**, 031301 (2012).

4. T. Wu, A. Bur, P. Zhao, K. P. Mohanchandra, K. Wong, K. L. Wang, C. S. Lynch, G. P. Carman, Giant electric-field-induced reversible and permanent magnetization reorientation on magnetoelectric Ni/(011)$[Pb(Mg_{1/3}Nb_{2/3})O_3]_{(1-x)}$–$[PbTiO_3]_x$ heterostructure. *Appl. Phys. Lett.* **98**, 012504 (2011).

5. Z. Wang, Y. Wang, H. Luo, J. Li, D. Viehland, Crafting the strain state in epitaxial thin films: A case study of $CoFe_2O_4$ films on Pb(Mg, Nb)O_3−$PbTiO_3$. *Phys. Rev. B* **90**, 134103 (2014).

6. M. Buzzi, R. V. Chopdekar, J. L. Hockel, A. Bur, T. Wu, N. Pilet, P. Warnicke, G. P. Carman, L. J. Heyderman, F. Nolting, Single domain spin manipulation by electric fields in strain coupled artificial multiferroic nanostructures. *Phys. Rev. Lett.* **111**, 027204 (2013).

7. A. Chen, Y. Wen, B. Fang, Y. Zhao, Q. Zhang, Y. Chang, P. Li, H. Wu, H. Huang, Y. Lu, Z. Zeng, J. Cai, X. Han, T. Wu, X.-X. Zhang, Y. Zhao, Giant nonvolatile manipulation of magnetoresistance in magnetic tunnel junctions by electric fields via magnetoelectric coupling. *Nat. Commun.* **10**, 243 (2019).

8. M. Liu, B. M. Howe, L. Grazulis, K. Mahalingam, T. Nan, N. X. Sun, G. J. Brown, Voltage-impulse-induced non-volatile ferroelastic switching of ferromagnetic resonance for reconfigurable magnetoelectric microwave devices. *Adv. Mater.* **25**, 4886–4892 (2013).

9. T. Nan, M. Liu, W. Ren, Z.-G. Ye, N. X. Sun, Voltage control of metal-insulator transition and non-volatile ferroelastic switching of resistance in VO_x/PMN-PT heterostructures. *Sci. Rep.* **4**, 5931 (2015).

10. B. Noheda, D. E. Cox, G. Shirane, J. Gao, Z.-G. Ye, Phase diagram of the ferroelectric relaxor $(1-x)PbMg_{1/3}Nb_{2/3}O_3-xPbTiO_3$. *Phys. Rev. B* **66**, 054104 (2002).

11. M. Davis, Picturing the elephant: Giant piezoelectric activity and the monoclinic phases of relaxor-ferroelectric single crystals. *J. Electroceram.* **19**, 25–47 (2007).

12. F. Li, L. Jin, Z. Xu, D. Wang, S. Zhang, Electrostrictive effect in $Pb(Mg_{1/3}Nb_{2/3})O_3$-$x$$PbTiO_3$ crystals. *Appl. Phys. Lett.* **102**, 152910 (2013).

13. J.-M. Hu, Z. Li, L.-Q. Chen, C.-W. Nan, High-density magnetoresistive random access memory operating at ultralow voltage at room temperature. *Nat. Commun.* **2**, 553 (2011).

14. S. Manipatruni, D. E. Nikonov, C.-C. Lin, T. A. Gosavi, H. Liu, B. Prasad, Y.-L. Huang, E. Bonturim, R. Ramesh, I. A. Young, Scalable energy-efficient magnetoelectric spin–orbit logic. *Nature* **565**, 35–42 (2019).

15. M. Boota, E. P. Houwman, M. Dekkers, M. D. Nguyen, K. H. Vergeer, G. Lanzara, G. Koster, G. Rijnders, Properties of epitaxial, (001)- and (110)-oriented $(PbMg_{1/3}Nb_{2/3}O_3)_{2/3}$-$(PbTiO_3)_{1/3}$ films on silicon described by polarization rotation. *Sci. Technol. Adv. Mater.* **17**, 45–57 (2016).

16. R. Keech, C. Morandi, M. Wallace, G. Esteves, L. Denis, J. Guerrier, R. L. Johnson-Wilke, C. M. Fancher, J. L. Jones, S. Trolier-McKinstry, Thickness-dependent domain wall reorientation in 70/30 lead magnesium niobate-lead titanate thin films. *J. Am. Ceram. Soc.* **100**, 3961–3972 (2017).

17. S. Pandya, J. Wilbur, J. Kim, R. Gao, A. Dasgupta, C. Dames, L. W. Martin, Pyroelectric energy conversion with large energy and power density in relaxor ferroelectric thin films. *Nat. Mater.* **17**, 432–438 (2018).

18. K. Lefki, G. J. M. Dormans, Measurement of piezoelectric coefficients of ferroelectric thin films. *J. Appl. Phys.* **76**, 1764–1767 (1994).

19. S. Trolier-McKinstry, P. Muralt, Thin film piezoelectrics for MEMS. *J. Electroceram.* **12**, 7–17 (2004).

20. P. B. Meisenheimer, S. Novakov, N. M. Vu, J. T. Heron, Perspective: Magnetoelectric switching in thin film multiferroic heterostructures. *J. Appl. Phys.* **123**, 240901 (2018).

21. G. P. Carman, N. Sun, Strain-mediated magnetoelectrics: Turning science fiction into reality. *MRS Bull.* **43**, 822–828 (2018).

22. H. Palneedi, V. Annapureddy, S. Priya, J. Ryu, Status and perspectives of multiferroic magnetoelectric composite materials and applications. *Actuators* **5**, 9 (2016).

23. J. Ma, J. Hu, Z. Li, C.-W. Nan, Recent progress in multiferroic magnetoelectric composites: From bulk to thin films. *Adv. Mater.* **23**, 1062–1087 (2011).

24. W. Gao, Y. Zhu, Y. Wang, G. Yuan, J.-M. Liu, A review of flexible perovskite oxide ferroelectric films and their application. *J. Mater.* **6**, 1–16 (2020).

25. S. H. Baek, J. Park, D. M. Kim, V. A. Aksyuk, R. R. Das, S. D. Bu, D. A. Felker, J. Lettieri, V. Vaithyanathan, S. S. N. Bharadwaja, N. Bassiri-Gharb, Y. B. Chen, H. P. Sun, C. M. Folkman, H. W. Jang, D. J. Kreft, S. K. Streiffer, R. Ramesh, X. Q. Pan, S. Trolier-McKinstry, D. G. Schlom, M. S. Rzchowski, R. H. Blick, C. B. Eom, Giant piezoelectricity on Si for hyperactive MEMS. *Science* **334**, 958–961 (2011).

26. R. Keech, L. Ye, J. L. Bosse, G. Esteves, J. Guerrier, J. L. Jones, M. A. Kuroda, B. D. Huey, S. Trolier-McKinstry, Declamped piezoelectric coefficients in patterned 70/30 lead magnesium niobate-lead titanate thin films. *Adv. Funct. Mater.* **27**, 1605014 (2017).

27. V. Nagarajan, A. Roytburd, A. Stanishevsky, S. Prasertchoung, T. Zhao, L. Chen, J. Melngailis, O. Auciello, R. Ramesh, Dynamics of ferroelastic domains in ferroelectric thin films. *Nat. Mater.* **2**, 43–47 (2003).

28. S. Bühlmann, B. Dwir, J. Baborowski, P. Muralt, Size effect in mesoscopic epitaxial ferroelectric structures: Increase of piezoelectric response with decreasing feature size. *Appl. Phys. Lett.* **80**, 3195–3197 (2002).

29. M. Wallace, R. L. Johnson-Wilke, G. Esteves, C. M. Fancher, R. H. T. Wilke, J. L. Jones, S. Trolier-McKinstry, In situ measurement of increased ferroelectric/ferroelastic domain wall motion in declamped tetragonal lead zirconate titanate thin films. *J. Appl. Phys.* **117**, 054103 (2015).

30. F. Griggio, S. Jesse, A. Kumar, O. Ovchinnikov, H. Kim, T. N. Jackson, D. Damjanovic, S. V. Kalinin, S. Trolier-McKinstry, Substrate clamping effects on irreversible domain wall dynamics in lead zirconate titanate thin films. *Phys. Rev. Lett.* **108**, 157604 (2012).

31. H. Greve, E. Woltermann, H.-J. Quenzer, B. Wagner, E. Quandt, Giant magnetoelectric coefficients in $(Fe_{90}Co_{10})_{78}Si_{12}B_{10}$-AlN thin film composites. *Appl. Phys. Lett.* **96**, 182501 (2010).

32. D. Viehland, M. Wuttig, J. McCord, E. Quandt, Magnetoelectric magnetic field sensors. *MRS Bull.* **43**, 834–840 (2018).

33. J. Irwin, S. Lindemann, W. Maeng, J. J. Wang, V. Vaithyanathan, J. M. Hu, L. Q. Chen, D. G. Schlom, C. B. Eom, M. S. Rzchowski, Magnetoelectric coupling by piezoelectric tensor design. *Sci. Rep.* **9**, 19158 (2019).

34. D. Lu, D. J. Baek, S. S. Hong, L. F. Kourkoutis, Y. Hikita, H. Y. Hwang, Synthesis of freestanding single-crystal perovskite films and heterostructures by etching of sacrificial water-soluble layers. *Nat. Mater.* **15**, 1255–1260 (2016).

35. J. Lee, C. H. Choi, B. H. Park, T. W. Noh, J. K. Lee, Built-in voltages and asymmetric polarization switching in $Pb(Zr,Ti)O_3$ thin film capacitors. *Appl. Phys. Lett.* **72**, 3380–3382 (1998).

36. S. Yokoyama, S. Okamoto, H. Funakubo, T. Iijima, K. Saito, H. Okino, T. Yamamoto, K. Nishida, T. Katoda, J. Sakai, Crystal structure, electrical properties, and mechanical response

of (100)−/(001)-oriented epitaxial $Pb(Mg_{1/3}Nb_{2/3})O_3$–$PbTiO_3$ films grown on $(100)_c SrRuO_3$‖(100)$SrTiO_3$ substrates by metal-organic chemical vapor deposition. *J. Appl. Phys.* **100**, 054110 (2006).

37. D. Fu, H. Taniguchi, M. Itoh, S. Koshihara, N. Yamamoto, S. Mori, Relaxor $Pb(Mg_{1/3}Nb_{2/3})O_3$: A ferroelectric with multiple inhomogeneities. *Phys. Rev. Lett.* **103**, 207601 (2009).

38. A. Kumar, J. N. Baker, P. C. Bowes, M. J. Cabral, S. Zhang, E. C. Dickey, D. L. Irving, J. M. LeBeau, Atomic-resolution electron microscopy of nanoscale local structure in lead-based relaxor ferroelectrics. *Nat. Mater.* **20**, 62–67 (2021).

39. H. Takenaka, I. Grinberg, S. Liu, A. M. Rappe, Slush-like polar structures in single-crystal relaxors. *Nature* **546**, 391–395 (2017).

40. J.-M. Hu, T. Yang, J. Wang, H. Huang, J. Zhang, L.-Q. Chen, C.-W. Nan, Purely electric-field-driven perpendicular magnetization reversal. *Nano Lett.* **15**, 616–622 (2015).

41. J.-M. Hu, T. Yang, K. Momeni, X. Cheng, L. Chen, S. Lei, S. Zhang, S. Trolier-McKinstry, V. Gopalan, G. P. Carman, C.-W. Nan, L.-Q. Chen, Fast magnetic domain-wall motion in a ring-shaped nanowire driven by a voltage. *Nano Lett.* **16**, 2341–2348 (2016).

42. M. G. Han, M. S. J. Marshall, L. Wu, M. A. Schofield, T. Aoki, R. Twesten, J. Hoffman, F. J. Walker, C. H. Ahn, Y. Zhu, Interface-induced nonswitchable domains in ferroelectric thin films. *Nat. Commun.* **5,** 4693 (2014).

43. D. J. Baek, D. Lu, Y. Hikita, H. Y. Hwang, L. F. Kourkoutis, Ultrathin epitaxial barrier layer to avoid thermally induced phase transformation in oxide heterostructures. *ACS Appl. Mater. Interfaces* **9**, 54–59 (2017).

44. J.-J. Wang, B. Wang, L.-Q. Chen, Understanding, predicting, and designing ferroelectric domain structures and switching guided by the phase-field method. *Annu. Rev. Mater. Res.* **49**, 127–152 (2019).

45. L.-Q. Chen, Phase-field method of phase transitions/domain structures in ferroelectric thin films: A review. *J. Am. Ceram. Soc.* **91**, 1835–1844 (2008).

46. H. Zhang, X. Lu, R. Wang, C. Wang, L. Zheng, Z. Liu, C. Yang, R. Zhang, B. Yang, W. Cao, Phase coexistence and Landau expansion parameters for a $0.70Pb(Mg_{1/3}Nb_{2/3})O_3$–$0.30PbTiO_3$ single crystal. *Phys. Rev. B* **96**, 054109 (2017).

47. J. J. Wang, X. Q. Ma, Q. Li, J. Britson, L.-Q. Chen, Phase transitions and domain structures of ferroelectric nanoparticles: Phase field model incorporating strong elastic and dielectric inhomogeneity. *Acta Mater.* **61**, 7591–7603 (2013).

48. Y. L. Li, S. Y. Hu, Z. K. Liu, L. Q. Chen, Effect of electrical boundary conditions on ferroelectric domain structures in thin films. *Appl. Phys. Lett.* **81**, 427–429 (2002).

49. J. F. Nye, *Physical Properties of Crystals: Their Representation by Tensors and Matrices* (Oxford Univ. Press, 1957), 134 pp.

50. M. J. Haun, E. Furman, S. J. Jang, L. E. Cross, Modeling of the electrostrictive, dielectric, and piezoelectric properties of ceramic $PbTiO_3$. *IEEE Trans. Ultrason. Ferroelectr. Freq. Control* **36**, 393–401 (1989).

51. R. E. Newnham, V. Sundar, R. Yimnirun, J. Su, Q. M. Zhang, Electrostriction: Nonlinear electromechanical coupling in solid dielectrics. *J. Phys. Chem. B* **101**, 10141–10150 (1997).

52. C. Tannous, J. Gieraltowski, The Stoner–Wohlfarth model of ferromagnetism. *Eur. J. Phys.* **29**, 475–487 (2008).

53. C. Kittel, Physical theory of ferromagnetic domains. *Rev. Mod. Phys.* **21**, 541–583 (1949).
